# Efficacy and the Safety of Granulocyte Colony-Stimulating Factor Treatment in Patients with Muscular Dystrophy: A Non-Randomized Clinical Trial

**DOI:** 10.3389/fneur.2017.00566

**Published:** 2017-10-26

**Authors:** Dorota Sienkiewicz, Wojciech Kułak, Bożena Okurowska-Zawada, Grażyna Paszko-Patej, Janusz Wojtkowski, Karolina Sochoń, Anna Kalinowska, Kamila Okulczyk, Jerzy Sienkiewicz, Edward McEachern

**Affiliations:** ^1^Department of Pediatric Rehabilitation, Medical University of Bialystok, Białystok, Poland; ^2^Białystok Technical University, Białystok, Poland; ^3^Medicine Bioscientific Research Faculty, Metro Health Medical Center Case Western Reserve, University School of Medicine, Cleveland, OH, United States

**Keywords:** granulocyte colony-stimulating factor, safety, efficacy, muscle strength, muscular dystrophy, children

## Abstract

**Introduction:**

The current standard treatment for patients with Duchenne muscular dystrophy (DMD) involves corticosteroids. Granulocyte colony-stimulating factor (G-CSF) induces the proliferation of satellite cells and myoblasts and, in turn, muscle regeneration. Beneficial effects of G-CSF were also described for skeletal muscle disorders.

**Aim:**

We assessed the safety and effects of using G-CSF to promote muscle strength in patients with DMD.

**Materials and methods:**

Inclusion criteria were as follows: patients aged 5–15 years with diagnosed with DMD confirmed by genetic test or biopsy. Fourteen patients were treated with steroids, and their use was not changed in this study. Diagnoses were confirmed by genetic tests: deletions were detected in 11 patients and duplications in 5 patients. Nineteen 5- to 15-year-old patients diagnosed with DMD—9 were in wheelchairs, whereas 10 were mobile and independent—completed an open study. Participants received a clinical examination and performed physiotherapeutic and laboratory tests to gage their manual muscle strength, their isometric force using a hand dynamometer, and aerobic capacity [i.e., 6-min walk test (6MWT)] before and after therapy. Each participant received G-CSF (5 µg/kg/body/day) subcutaneously for five consecutive days during the 1st, 2nd, 3rd, 6th, and 12th month. Laboratory investigations that included full blood count and biochemistry were performed. Side effects of G-CSF treatment were assessed during each visit. During each cycle of G-CSF administration in the hospital, rehabilitation was also applied. All patients received regular ambulatory rehabilitation.

**Results:**

The subcutaneous administration of G-CSF improved muscle strength in participants. We recorded a significant increase in the distance covered in the 6MWT, either on foot or in a wheelchair, increased muscle force in isometric force, and a statistically significant decrease in the activity of the muscle enzyme creatine kinase after nearly every cycle of treatment. We observed no side effects of treatment with G-CSF.

**Conclusion:**

Our findings suggest that G-CSF increases muscle strength in patients with DMD, who demonstrated that G-CSF therapy is safe and easily tolerable.

## Introduction

Muscular dystrophies comprise about 30 disease entities (1, 2). Currently, there is no effective therapeutic tools for muscular dystrophies so far. The present gold standard of treating patients with Duchenne muscular dystrophy (DMD) is corticosteroids that cause the course of the disorder to slow (3).

Duchenne muscular dystrophy is a hereditary X-linked neuromuscular disorder caused by mutations in the dystrophin gene, with a prevalence of 1:5,000 male newborns (4). It is caused by the lack of functional dystrophin protein due to nonsense mutations in the DMD gene, deletions (small), or duplications (small). These mutations decrease synthesis of dystrophin in muscles. Dystrophin protect muscle cells from damage (5). DMD is a severe muscle dystrophy with muscle weakness and causes loss of motor function, heart and respiratory failure and, eventually, death (6). The first symptoms of DMD can be seen before 5 years but a large proportion of patients’ loss of independent walking beyond 12 years (7). Diagnosis of and therapy for patients with muscular dystrophy should be initiated as early as possible to prevent motor function delay (8, 9).

Researchers are searching for an effective therapeutic approach, including gene and stem cell-based therapies. Beneficial effects of granulocyte colony-stimulating factor (G-CSF) were also described for skeletal muscle disorders (10, 11). Stratos and his coworkers (10) found that after a blunt muscle injury in animals, administration of G-CSF increased muscular regeneration by satellite cell proliferation and decreased apoptosis. Also, Hara et al. (11) showed that G-CSF and its receptor play important roles in muscle development and regeneration.

Granulocyte colony-stimulating factor is a hematopoietic cytokine, widely used for mobilization of hematopoietic stem cells from bone marrow and to treat neutropenia after chemotherapy (12). In 2014, G-CSF was tested in the animal model of DMD, the mdx mouse (13). It was found that treated mdx mice had a higher number of normal muscle fibers compared with untreated mdx mice. Treated mice had 62% of normal muscle fibers and reduced inflammation.

Granulocyte colony-stimulating factor induces (directly or through the increase in circulating stem cells) the production of many growth factors (for example: insulin-like growth factor 1, epidermal and transforming growth factors, and cytokines) and may have other methods of action on the system of musculature, vessels, and nerves yet to be described (14). The mechanism of action of G-CSF may also include enhanced successful divisions of satellite stem cells as recently reported by Canadian researchers (15).

To date and to the best of our knowledge, there have not been any published studies reporting the use or effects of G-CSF in children with muscular dystrophies.

The purpose of this open trial is to evaluate the efficacy, and we assessed the safety and effects of G-CSF on muscle strength in patients with DMD.

## Materials and Methods

### Study Design

A prospective, non-randomized clinical trial assessed the efficacy and safety of G-CSF treatment in patients with muscular dystrophy.

### Participants

**Figure 1:**
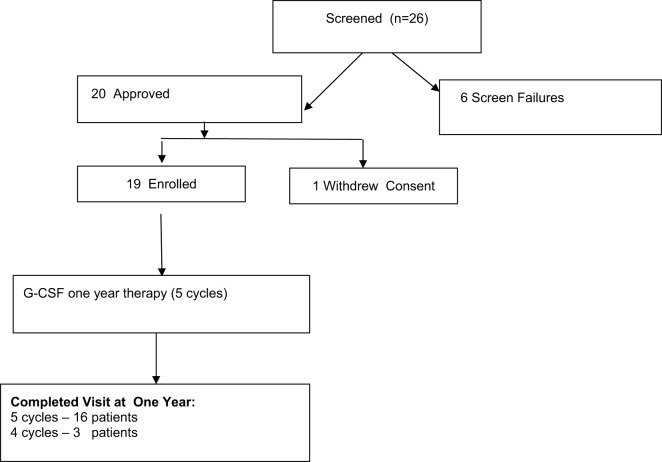
Study flowchart of granulocyte colony-stimulating factor treatment 1-year therapy of patients with muscular dystrophy. Screening, enrollment, and follow-up.

We enrolled 26 patients with muscular dystrophies under care of our department. Details are shown in Figure 1. Six patients were screen failures: three patients with Becker muscular dystrophy and three patients with facioscapulohumeral dystrophy.

Inclusion criteria were as follows: patients aged 5–15 years with diagnosed with DMD confirmed by genetic test or biopsy. Fourteen patients were treated with steroids, and their use was not changed in this study. Diagnoses were confirmed by genetic tests: deletions were detected in 11 patients and duplications in 5 patients.

Nine children (47.4%) were wheelchair-bound, and the others (52.6%) were mobile and self-independent. Details are shown in Table 1.

**Table 1:** Characteristics of the study population.

Age—range, mean ± SD	5–15 years (9.4 ± 2.6)
Sex	M 19 (100%)
Type of dystrophy	DMD 19 (100%)
Wheelchair	(+) 9 (47.4%)
Self-independent	(−) 10 (52.6%)
Corticosteroids therapy	(+) 14 (73.7%)
	(−) 5 (26.3%)
G-CSF treatment courses (each one: 5 μg/kg/day × 5 μg/kg/day)	5 courses 16 (84.2%)
	4 courses 3 (15.8%)
Genetics tests DMD	11 patients—deletion in exon; 2–4; 8–11; 17; 45; 44–47; 45–46; 49–50; 49–54; 51; 46–47
	4 patients—duplication in exon: 2–9; 8–48; 50–54; 53
	1 patient—punctuation mutation 16

*G-CSF, granulocyte colony-stimulating factor; DMD, Duchenne muscular dystrophy*.

### Outcome Measures

We expected that G-CSF (5 μg/kg/body/day) administration subcutaneously in patients with muscular dystrophies during the 1st, 2nd, 3rd, 6th, and 12th month increase in the passed distance in the 6-min walk test (6MWT) by feet or wheelchair, and an increase in muscle force compared with baseline would be observed.

### Assessment

Disease course was evaluated clinically by neurological assessments (16). Manual muscle testing (Lovett test) of the upper and lower limbs, isometric force with the hand dynamometer, and 6MWT (17) were measured before and after therapy.

### Safety

Laboratory investigations that included full blood count, biochemistry [CRP, creatinine, glucose, electrolytes—Na, K, Cl, Ca, Mg, fibrinogen, partial thromboplastin time, prothrombin time, creatine kinase (CK), and urine (Laboratory of the Medical University Children Hospital)] were performed. Blood was collected before G-CSF administration and on the fifth day of each treatment cycle. The assessment of hematopoietic stem/progenitor cells (CD34+), endothelial progenitor cells (CD34+ CD133+ CD309), monocyte subsets (CD14, CD16) was performed using flow cytometry in 11 patients in previous study (18).

Abdominal ultrasonography with a spleen measurement was done before and after G-CSF administration. Electrocardiographic records were also performed. Side effects of G-CSF treatment were assessed during each visit.

### G-CSF Administration

**Figure 2:**
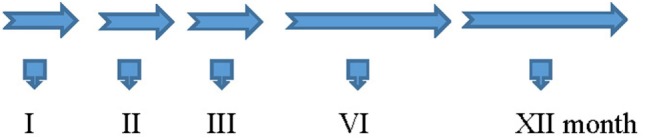
The treatment protocol schema.

Granulocyte colony-stimulating factor (5 μg/kg/body/day) was administrated subcutaneously for five consecutive days during the 1st, 2nd, 3rd, 6th, and 12th month (Amgen, USA). We decided to apply half of G-CSF dose using for oncology treatment in children because of safety requirements. During each cycle of G-CSF administration in the hospital, rehabilitation was also applied. All patients received regular ambulatory rehabilitation. The treatment protocol schema is shown on Figure 2.

### G-CSF 5 μg/kg/day, sc—Five Doses

1. Interview and physical examination
2. Physiotherapeutic examination: 6MT, Lovett Test, Hand Dynamometer Test
3. Laboratory tests
4. Ultrasound of abdominal cavity with liver and spleen measurement
5. ECG
6. Spirometry

### Ethics

Ethical approval for the study was obtained from the ethic committee of the Medical University of Bialystok (R-I-002/375/2013). Written informed consent was obtained from the patients and parents before participation.

We have started our study in March 1, 2013, and we recruited patients from this time. We have registered (June 23, 2016) our clinical trial at website of Clinicaltrials.gov to increase significance of the trial. The study Clinicaltrials.gov Identifier: NCT02814110.

### Statistical Analysis

All statistical analysis was performed with SPSS package 15.0. Results are presented as mean values ± SD. Parametric paired *t*-test was applied to compare difference in time. The critical level for all tests of significance was *p* < 00.05.

## Results

### Patients

Of the 19 patients, 16 (84.2%) completed the study. Patients were 5–15 years old, with a mean age (9.4 ± 2.6). Between March 2013 and February 2017, a total of 19 patients were treated with G-CSF. Sixteen patients completed five courses of the G-CSF treatment, and three boys ended earlier—after the fourth course (one parent refused because of a lack of clinical improvement).

### Results of Investigation

No side effects after G-CSF administration were reported by patients. White blood cell count increased at fifth day after each G-CSF administration as a reaction to drug application. Red blood count, platelets, CRP, creatinine, glucose, electrolytes—Na, K, Cl, Ca, Mg, fibrinogen, partial thromboplastin time, and prothrombin time were in the normal range. We observed a statistically significant decrease of the activity of muscle enzyme—CK after each cycle of treatment (Table 2).

**Table 2:** Changes in creatine kinase (CK) activity in 19 patients in ambulant and non-ambulant patients with Duchenne muscular dystrophy in each cycle of treatment.

CK		Mean	SD	*p*-Value
Cycle 1	1	11,385.1	7,423.7	0.001
	2	5,146.7	3,376.8	
Cycle 2	1	7,446.6	5,234.0	0.001
	2	4,674.3	3,843.8	
Cycle 3	1	8,774.9	8,650.1	0.002
	2	3,963.1	3,449.7	
Cycle 4	1	9,139.8	8,353.8	0.007
	2	4,620.3	4,082.4	
Cycle 5	1	10,019.8	10,868.7	0.010
	2	4,309.5	4,049.1	

*1, CK activity before treatment; 2, CK activity after treatment*.

*Cycle 1, 1st month; Cycle 2, 2nd month; Cycle 3, 3rd month; Cycle 4, 6th month; Cycle 5, 12th month*.

In an ultrasound examination, the spleen size was normal during treatment. Electrocardiographic records did not differ significantly. We evaluated the effect of G-CSF treatment on the muscle strength and physical activity of the patients by performing these tests: (i) 6MWT to evaluate walking distance or moving wheelchair distance during 6 min, (ii) isometric force with hand dynamometer, and (iii) manual muscle testing (Lovett Test).

- We found significant increase of distance in 6MWT between baseline and first, second, third, and fourth cycle in ambulant patients with DMD. We observed also significant increase of distance between baseline first and third cycle in wheelchair-dependent patients with DMD. Details are shown in Table 3.
- We also found significant increase of muscle strength in right hand in ambulant patients with DMD after the first, second, third, fourth, and fifth cycle of G-CSF treatment in comparison with the baseline. Only significant increase of muscle strength in left hand was noted after the fifth cycle.
- Significant increase of muscle strength in right and left hand was noted in wheelchair-dependent patients with DMD only after the fourth cycle of G-CSF treatment in comparison with the baseline. See Table 4.
  The minimal increase of muscle force was found (Lovett test), but not statistically significant. Details are not shown.
- We found significant (*p* = 0.001) increase of forced vital capacity in patients with DMD after fifth cycle of G-CSF treatment (1.69 ± 0.44 L) in comparison with the baseline (1.54 ± 0.43 L). We also noted significant (*p* = 0.032) increase of forced expiratory volume in 1 s (FEV1) in patients with DMD after fifth cycle of G-CSF treatment (1.53 ± 0.40 L) in comparison with the baseline (1.42 ± 0.37 L).

**Table 3:** Effect of granulocyte colony-stimulating treatment on 6-min walk test between baseline and each other cycle in ambulant and non-ambulant patients with Duchenne muscular dystrophy.

Cycle number	Baseline—0	Mean (m)	*N*	SD	*p*-Value
	After treatment number cycle 1–5				
**Ambulant patients**					
1	0	302.2	10	96.3	**0.001**
	1	337.7	10	108.1	
2	0	302.2	10	96.3	**0.005**
	2	364.8	10	113.9	
3	0	302.2	10	96.3	**0.039**
	3	355.3	10	121.9	
4	0	302.2	10	96.3	**0.028**
	4	373.5	10	143.0	
5	0	309.1	9	99.4	0.193
	5	353.4	9	149.9	
**Wheelchair-dependent patients**					
1	0	158.3	9	116.2	**0.046**
	1	170.6	9	121.3	
2	0	170.6	9	121.3	0.060
	2	207.6	9	110.5	
3	0	207.6	9	110.5	**0.004**
	3	229.6	9	98.2	
4	0	229.6	9	98.2	0.262
	4	216.9	9	75.8	
5	0	213.9	7	75.0	0.600
	5	162.0	7	80.3	

*N, number patients*.

**Table 4:** Effect of granulocyte colony-stimulating factor treatment on muscle strength in hand dynamometer test (in kg) between baseline and each other cycle in ambulant and non-ambulant patients with Duchenne muscular dystrophy.

Hand	Cycle number	Baseline—0	Mean	*N*	SD	*p*-Value
		After treatment number cycle 1–5				
**Ambulant patients**						
Right	1	0	3.62	10	2.30	**0.049**
		1	4.37	10	2.54	
	2	0	3.58	9	2.44	**0.008**
		2	4.89	9	2.71	
	3	0	3.62	10	2.30	**0.001**
		3	5.26	10	2.72	
	4	0	3.62	10	2.30	**0.001**
		4	5.62	10	2.72	
	5	0	3.88	9	2.28	**0.001**
		5	6.28	9	2.36	
Left	1	0	3.86	10	2.31	0.657
		1	3.98	10	2.32	
	2	0	3.68	9	2.37	0.087
		2	4.42	9	2.08	
	3	0	4.01	9	2.39	0.067
		3	4.99	9	2.56	
	4	0	3.86	10	2.31	0.115
		4	5.73	10	3.14	
	5	0	4.18	9	2.20	**0.024**
		5	5.53	9	2.83	
**Non-ambulant patients**						
Right	1	0	2.14	9	1.57	0.184
		1	1.95	9	1.47	
	2	0	2.41	8	1.44	0.875
		2	2.48	8	1.70	
	3	0	2.14	9	1.57	0.285
		3	2.67	9	1.83	
	4	0	2.14	9	1.57	**0.025**
		4	3.21	9	2.14	
	5	0	1.96	7	1.70	0.360
		5	2.64	7	1.67	
Left	1	0	2.14	9	1.69	0.435
		1	1.98	9	1.50	
	2	0	2.40	8	1.59	0.198
		2	2.84	8	1.80	
	3	0	2.14	9	1.69	0.152
		3	2.72	9	1.97	
	4	0	2.14	9	1.69	**0.003**
		4	3.20	9	1.93	
	5	0	2.01	7	1.88	0.221
		5	2.83	7	1.79	

*N, number of patients*.

## Discussion

As an effect of G-CSF treatment in our study group, we observed a significant increase of distance in 6MWT in the first 6 months of therapy. After the next half-a-year break from treatment, we found a decrease in the boys’ performance, yet an effect that was still statistically significantly better than when the trial began. This decrease is possibly due to the longer time periods between administration (6 months between course 4 and 5), whereas the first administrations were 1 month apart. The increase of muscle strength testing by hand dynamometer was less spectacular but also detected. In laboratory tests, we observed a decrease in the activity of muscular enzymes.

Although stem-cells therapy is still in its beginning stages, there are more and more observations of the positive effects of it and G-CSF treatment in patients with neurological diseases (19). G-CSF increase the proliferation of satellite cells, with transformation into myotubes and muscle fibers, and promote of muscle regeneration (11, 20). These results may point to the general activation of the entire system of cellular regulation rather than a specific target.

It has been shown that G-CSF decreases inflammatory processes and acts positively on peripheral nerve regeneration during the course of muscular dystrophy. This effect was observed in Simões’s study on mdx mice (13). The authors suggest that besides nerve regeneration, G-CSF promotes a favorable microenvironment for axonal regeneration, thereby slowing the progression of DMD. The other authors also indicated that on animal models, G-CSF is important for skeletal myocyte development and regeneration (11, 21).

There is a growing body of evidence that monocytes/macrophages play an important role in muscle regeneration. Macrophages MI (pro-inflammatory cells) are involved in immune activation, phagocytosis, and muscle cell lysis. Macrophages MII exert anti-inflammatory properties and participate in the vascularization process. This population is able to support muscle cell regeneration by including satellite cell proliferation and tissue revascularization (22). To find exact mechanisms underlying beneficial effects of G-CSF in patients with neuromuscular disorders, Eljaszewicz et al. (18) used flow cytometry to quantitate numbers of CD34+ cells, endothelial progenitor cells, and different monocyte subsets in the peripheral blood of the patients treated with repetitive courses of G-CSF administration. The observed effect was an inducement of efficient mobilization of the abovementioned cells, including cells with proangiogenic potential.

As to the administration of G-CSF in clinical trials, researchers have found neurological improvement in motor and sensory functions in adult patients with worsening symptoms of compression myelopathy (23). These findings were in accordance with Kato’s (24) open-label, single-center clinical trial. In this study, 17 patients with compression myelopathy underwent intravenous administration of G-CSF (10 μg/kg/day) for five consecutive days. They observed a reduction in pain without any adverse events during or after G-CSF administration. Similar beneficial effects on spinal pathology—acute spinal cord injury—were observed by Inada (25).

In this study, we found significant decrease in the activity of muscle enzymes CK after each cycle of treatment and a stability of this level until 6 months of observation. After 12 months, a decrease of this enzyme activity was also observed, and it was statistically significant. In patients with muscular dystrophy the loss of muscle mass is observed, and, after an increase, a decrease of CK activity in the blood is noted. In our investigation, the situation seems to have been different. The abovementioned changes in laboratory parameters were accompanied by an augmentation of passed distance—by foot or on wheelchair and an increase in muscle force detected by dynamometry measurement.

Furthermore, it is known that with G-CSF-induced mobilization of many trophic cells—endothelial progenitor cells, monocyte subsets, and cells with proangiogenic potential—there is the suggestion of possible anti-inflammatory effect, mobilization of existing satellite cells, improvement of local vascularity, and cytoprotection. We think that in DMD patients, the effect of G-CSF treatment comes from its positive impact on the regeneration and maintenance of the muscle fibers.

Because the effects of therapy diminished after a 6-month break from G-CSF application, it seems to be more beneficial to administer a drug each 4 months. Our report has several limitations and strengths that are shown in Table 5.

**Table 5:** Cons and pros granulocyte colony-stimulating factor therapy in patients with Duchenne muscular dystrophy.

Pros	Cons
Long-term study	Open-label study—no control group
Objective functional testes	Small number of study group
Qualification patients with different types of dystrophy and at every age	Not a homogeneous group of study subjects
Lack of disqualification because the type of genome mutation	Different functional states of patients (independent or wheelchair dependent)
Well-tolerated therapy	Patients with or without steroids therapy

The major limitation is that this was an open study. Patients in different stages of disease, with or without steroid therapy were treated. In a qualitative evaluation, both medical staff and parents observed that children were more active, they had better mood, appetite, their state of balance and precise movements improved.

New genetic-based therapies in patients with DMD are promising. Exon skipping is a therapeutic approach for DMD. This method uses antisense oligonucleotides (AONs) to modulate pre-mRNA splicing of dystrophin transcripts to restore the disrupted DMD reading frame (26). Recently, eteplirsen, the AON targeting exon 51 became the first of its class to be approved by the United States regulators (Food and Drug Administration) for the treatment of DMD (27). This therapy is promising because it corrects the reading frame of the dystrophin-encoding gene and restores protein expression, resulting in the conversion of DMD to a clinically milder form.

## Conclusion

Our study shows that G-CSF therapy is safe and well tolerated by the patients. We showed statistically significant increase in 6MWT and muscle strength, due to G-CSF administration. Significant decrease of CK after each of G-CSF treatment cycle was seen. Our data suggest that G-CSF increases muscle strength in children and adolescents with muscular dystrophy. We recommend further studies to address this proposition, as well as the mechanism of action identified by the Canadian research, in that the effects could be both to increase the number of viable (satellite, nerve, and other) stem cells and growth factors, and also to increase the number of successful divisions that occur. The collective effect of these two features may well explain the significant collective improvements reflected in our data.

## Ethics Statement

Ethical approval for the study was obtained from the ethic committee of the Medical University of Bialystok (R-I-002/375/2013). Informed consent was obtained from the patient and parents before participation.

## Author Contributions

DS, WK, BO-Z, and GP-P contributed equally to this work, study conception, data analysis, data collection, and paper writing; JW, KS, AK, and KO made physiotherapeutic tests and data collection; JS made statistics; EM revised the paper and made remarks.

## Conflict of Interest Statement

The authors declare that the research was conducted in the absence of any commercial or financial relationships that could be construed as a potential conflict of interest. The reviewer RJ and handling editor declared their shared affiliation.
